# Pathology in General Practice

**Published:** 1907-02-09

**Authors:** 


### PATHOLOGY IN GENERAL PRACTICE.

No branch in medicine has advanced in recent years with such leaps and bounds as pathology, and its importance in clinical diagnosis cannot be too strongly insisted upon. Yet granting all this, how few practitioners in general practice are able to perform the simplest details, such as counting the corpuscles of the blood, estimating the hæmoglobin, searching a blood film for malarial parasites, staining for tubercle bacilli, staining for the gonococcus, examining and staining deposits from urine, to mention only a few of the very simplest procedures. To meet the demand for this kind of work, numerous private individuals and institutions, such as laboratories, have sprung up. The practitioner sends his samples here, pays a charge, and the results are sent him in due course. The more he does this the more he forgets how to do the thing himself, till eventually he relies entirely on this means of arriving, in many cases, at his diagnosis. Now how far is this right or wrong? Before coming to any definite decision one must glance for a moment at what the term pathology nowadays really means. It includes, for example, the subjects of bacteriology, protozoology, helminthology, hæmatology, morbid anatomy, gross and microscopical, and tropical pathology, the latter not an entity of its own but a mixture of all the foregoing plus an extended knowledge of entomology. The range, therefore, is a vast one, so vast, for example, that specialists have arisen on all its different component parts, and the general practitioner may well be pardoned if he exclaims: "How can I with my clinical work and practice be expected to be an expert on all those subjects?" His contention is perfectly justifiable as far as being an expert is concerned, but on the other hand, though not an expert, he should and ought to know many of the simple technical procedures necessary to demonstrate ordinary truths culled from the different special branches of which pathology is made up. A practical knowledge of the work required can easily be obtained at any of the post-graduate teaching associations, many of which have courses specially framed for this need, and books, variously styled clinical bacteriology or clinical bacteriology and hæmatology, give very good and accurate accounts of the various methods. The younger practitioners and those who have just recently qualified have, of course, learned those methods in their courses at their medical schools, and as far as they are concerned, a little practice is all that is necessary to keep them from forgetting the details when they commence the task of earning their daily bread. Allowing, then, that the ordinary man should be able to do his simple examinations, such as staining for tubercle, examining urines, etc., himself, there still remain a class of more complicated technical procedures which require incubators and a laboratory to be at one's disposal. Such, for example, are the Widal's reactions for typhoid and Malta fever and the recent works on opsonins and the opsonic index. It is, of course, impossible for everyone to have incubators where the cultures can be grown and put through the necessary sub-culturing, so this branch of clinical diagnosis must still be done in laboratories. We thus see the rights and the wrongs of the question under discussion when looked at from this point of view; but there are other issues which still have to be considered. The most important of these is the gradual separation of clinical from pathological work which results from the system of the practitioner relying on someone else to do a share of his work; and this separation is a most undesirable one and often leads to serious mistakes being perpetrated.

The value of all this new work is great when taken in conjunction with the clinical phenomena, but when used alone it may, as we have just stated, be extremely dangerous and misleading. Examples are so numerous that it is almost unnecessary to mention any here; all must know the common one of a small piece of the edge of a tumour being snipped off and sent to the pathologist for examination. On this examination a diagnosis is expected to be made; yet how fallacious this may be. The sections may show nothing very definite, say, with the exception of some chronic inflammatory changes, and a return to this effect is given, while all the time the patient may be suffering from true cancer, the reason of this not being detected being that by mischance the piece selected has been taken from the outside of the growth and not from the real diseased part at all. Again, only to quote another example, a blood from a real case of enteric fever may be taken early, before the serum reaction has developed, and sent to the laboratory; the result negative is returned, and the probability is that the practitioner treats the case as something else, fully believing now that it is not typhoid. Now, if the case had been followed carefully clinically and the Widal's test used several times during the course of the illness as an adjunct to corroborate the diagnosis only, such a mistake would not occur. Another rather important point is that when work is going at a high pressure in the institutions mentioned, laboratory attendants are often called upon to do some of it, and though those individuals are quite competent in most cases, they are often careless and do not appreciate the gravity of what the examination means. This undoubtedly accounts for many of the peculiar results that are obtained and has made some individuals so sceptical that they are in the habit of sending portions of the same specimens or fluids to one or two different places at the same time. One case of a physician illustrates this forcibly. He sent a blood serum to be tested for one of two diseases to three different laboratories and got back three different results—namely, that neither disease was present, that one was present, and from the third that the other was present. In a similar manner a urine known to be diabetic was returned as no sugar present, this being due to some other samples getting mixed up with it, and some individual must have got a surprise when he got the return for a urine, say, which he was having examined for casts, as full of sugar. Mistakes like this are regrettable, and it only shows that no one is infallible, but that is not the point. The point is that the two branches should not be divorced like this but should run in harmony together and then most of those mistakes could not occur, or if they did would be of no great importance. The general practitioner should most certainly make a point of doing his simple routine work himself, and in some subsequent articles the commoner methods will be explained to show how simple and easy of application they are.

